# Outcome of Patients With Differentiated Thyroid Cancer Treated With 131-Iodine on the Basis of a Detectable Serum Thyroglobulin Level After Initial Treatment

**DOI:** 10.3389/fendo.2019.00146

**Published:** 2019-03-12

**Authors:** Michele Klain, Leonardo Pace, Emilia Zampella, Teresa Mannarino, Simona Limone, Emanuela Mazziotti, Giovanni De Simini, Alberto Cuocolo

**Affiliations:** ^1^Dipartimento di Scienze Biomediche Avanzate, Università degli Studi di Napoli Federico II, Napoli, Italy; ^2^Dipartimento di Medicina, Chirurgia ed Odontoiatria Scuola Medica Salernitana, Università Degli Studi di Salerno, Salerno, Italy

**Keywords:** differentiated thyroid carcinoma, ^131^I empiric therapy, prognosis, thyroglobulin, whole body scan

## Abstract

**Background:** In patients with differentiated thyroid cancer (DTC) and raising serum thyroglobulin (Tg) after total or near-total thyroidectomy and ^131^I remnant ablation an empiric ^131^I therapy may be considered. However, outcome data after empiric therapy in did not show a clear evidence of improved survival. We assessed the efficacy of such empiric ^131^I therapy in patients with DTC and evaluated the long-term outcome.

**Methods:** A total of 100 patients with DTC showing raised Tg level during follow-up after thyroidectomy and ^131^I ablation were treated with a further ^131^I therapy (6.1 ± 1.7 GBq). Whole-body scan (WBS) was performed 5–7 days after therapy. Tg value at 12 months after ^131^I therapy was considered as an indicator of treatment response: ≤1.5 ng/ml complete remission (CR), >50% decrease partial remission (PR), higher than pre-therapy progression disease (PD), all other cases stable disease (SD). Patients were followed-up for 96 ± 75 months.

**Results:** After 12 months, 62% of patients were in CR, 16% in PR, 8% in SD, and 14% in PD. WBS was positive in 41% of patients and negative in 59% (*P* = NS). Among patients with local recurrences at WBS 89% showed either CR or PR, while 71% of patients with distant metastases were in SD or PD (*P* < 0.001). Distant metastases at WBS (*P* < 0.05), CR (*P* < 0.0001), and CR + PR (*P* < 0.0001) were predictors of both progression free survival and overall survival.

**Conclusion:** There is a beneficial effect of ^131^I therapy on outcome of patients with DTC treated on the basis of elevated Tg value. In these patients, survival is affected by achievement of CR or PR at 12 months evaluation after ^131^I therapy and by the presence of distant metastases at WBS.

## Introduction

Follow-up of patients with differentiated thyroid cancer (DTC) after total or near-total thyroidectomy and ^131^I remnant ablation is performed by assessment of thyroglobulin (Tg) levels, ultrasonography, and ^131^I diagnostic whole-body scan (d-WBS). The most reliable parameter for the detection of tumor recurrence is a raised Tg level, which suggests persistence or recurrence of viable tumor tissue (1–5). In these patients an empiric ^131^I therapy may be considered (6), although there is still no agreement on the cutoff value of serum Tg above which a patient should be treated with an empiric ^131^I dose. It has been reported that patients with stimulated serum Tg ≥ 5 ng/ml are unlikely to have a decline without therapy, while a high rate of subsequent structural recurrence has been reported (7, 8). In these patients, administration of high activity of ^131^I would have both a diagnostic and therapeutic intent. Actually, a decrease in Tg levels after empiric ^131^I therapy has been demonstrated in 56–63% of such patients and in 61% of them post-therapy ^131^I whole-body scan (t-WBS) was positive (9, 10). Yet, data on outcome after empiric therapy in this setting did not show a clear evidence of improved survival (11–13).

Therefore, the aims of this study were to assess the efficacy of a high-dose ^131^I therapy administered only on the basis of a raised serum Tg level in patients with DTC after initial treatment and to evaluate the long-term outcome.

## Materials and Methods

### Study Population and Design

**Figure 1:**
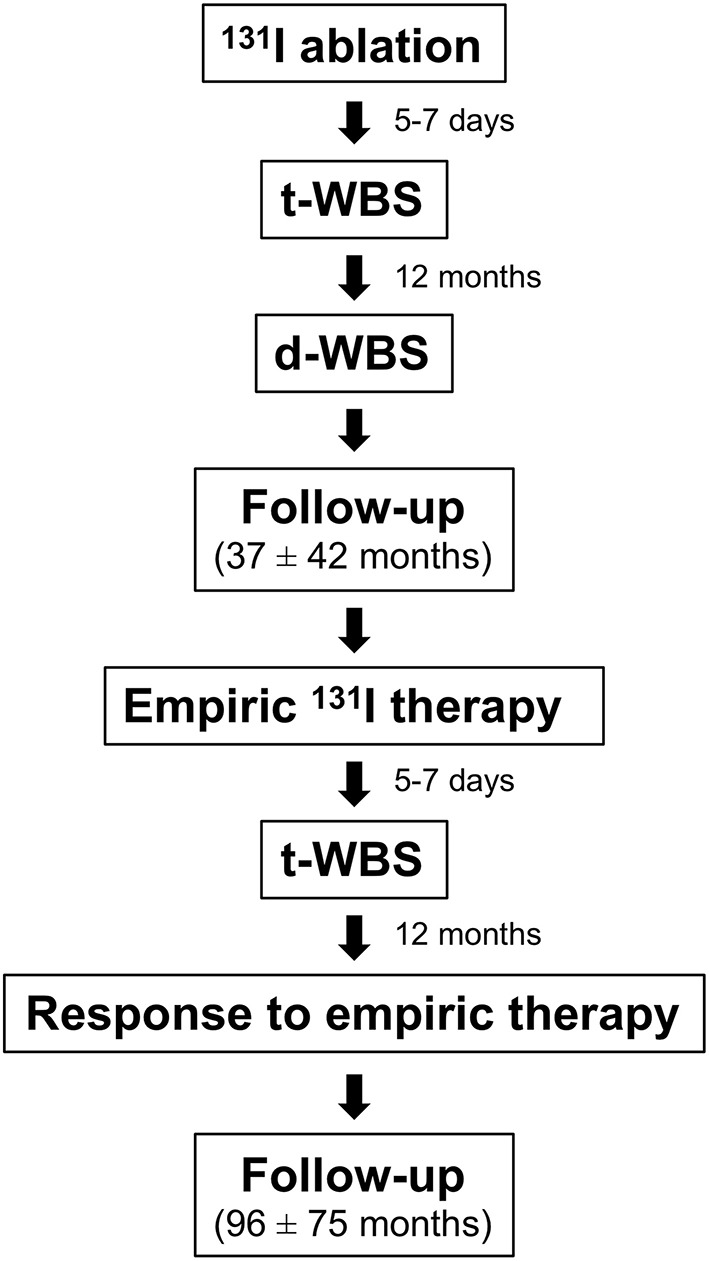
Outline of the study protocol.

Among 1,115 patients with DTC treated with total or near-total thyroidectomy followed by ^131^I ablation between 1998 and 2006, those (*n* = 100) showing raised (i.e., >5 ng/ml) serum Tg level during follow-up were included in this retrospective study. The study protocol is outlined in Figure 1. After ^131^I ablation, L-thyroxine therapy for TSH suppression was performed in all patients according to the American Thyroid Association (ATA) guidelines (6). Results of ^131^I ablation were evaluated after 12 months by Tg assessment off L-thyroxine therapy, d-WBS and neck ultrasound. All patients underwent d-WBS while off L-thyroxine therapy for 3 weeks. Imaging was performed 48 h after administration of 250 MBq of ^131^I using a dual-head gamma camera (E.CAM, Siemens Medical Systems, Hoffman Estates, IL, USA) equipped with a high-energy, general purpose collimator and connected with a dedicated computer system. Exclusion criteria were: positive serum anti-Tg antibody and a d-WBS scan 12 months after ^131^I ablation demonstrating thyroid remnant or secondary lesions. Hence, all patients showed an excellent response after ablation (6). For empiric ^131^I therapy, L-thyroxine was discontinued for 20-30 days until the TSH augmented (≥25–30 mIU/l). At that time, Tg concentration was measured and ^131^I was administered (6.1 ± 1.7 GBq). Five to seven days later, t-WBS was performed.

Since all patients had a negative d-WBS 12 months after ^131^I ablation, the presence of tracer uptake in the cervical region at t-WBS performed after empiric ^131^I therapy was considered as local recurrence, while uptake in other regions was considered as distant metastases. The presence of local recurrence and distant metastases was confirmed by additional imaging procedures such us ultrasound, computed tomography, and/or bone scintigraphy, as appropriate.

Response to ^131^I empiric therapy was evaluated by assessing the serum Tg level 12 months after empiric therapy. In particular, a Tg value ≤1.5 ng/ml was considered as complete remission (CR), a decrease >50% as partial remission (PR), a level higher than the pre-therapy value as progression disease (PD), while all other cases were considered to be in stable disease (SD) (14). Thereafter, patients were followed by Tg level determinations (on L-thyroxine and off L-thyroxine therapy, as requested) every 6–12 months and imaging procedures as appropriate, with a mean follow-up of 96 ± 75 months (range 12–276 months). Disease status was recorded at each evaluation time point. Progression free survival (PFS) was measured from the date of empiric ^131^I therapy to the first observation of progressive disease, relapse, need for additional therapy (i.e., ^131^I therapy or surgery), or death. Relapse of disease was defined as evidence of disease during follow-up after empiric ^131^I therapy by histological or imaging procedures, as appropriate, in patients with proven DTC and stimulated Tg levels > 2 ng/ml (15). Patients last known to be alive and progression free were censored at date of last contact. Overall survival (OS) was measured from the date of registration to the date of death.

### Statistical Analysis

MedCalc Statistical Software version 13.1.2 was used for statistical analysis (MedCalc Software bvba, Ostend, Belgium; http://www.medcalc.org; 2014). Continuous data were tested for normal distribution by the D'Agostino-Pearson test. Data were expressed as mean ± standard deviation, median and range or as proportions, as appropriate. Differences among groups were analyzed by analysis of variance, Kruskal-Wallis test, or by chi square analysis, as appropriated. Univariate and multivariate logistic analyses were used to evaluate significant determinants of response to 131-I therapy. Univariate and multivariate analyses of clinical and imaging variables were performed using Cox proportional hazards regression. Only significant variables at univariate analysis were included in the multivariate model. Survival analysis was performed using Kaplan-Meier method and log-rank test. Survivors were censored at the time of last clinical control. A *P* < 0.05 was considered statistically significant.

## Results

Among 100 patients (68 women and 32 men; mean age 46 ± 16 years, range 20–82 years) enrolled, 16 had follicular cancer, and 84 papillary cancer. At initial staging, after surgery and ^131^I ablation, 60 patients were in stage I, 10 in stage II, 23 in stage III, and 7 in stage IVa. According to the ATA risk stratification system, 43 patients were in low, 41 in intermediate, and 16 in high-risk group. t-WBS performed at ^131^I ablation was negative in 5 patients and showed tracer uptake in the cervical region (i.e., thyroid remnant) in 95 patients. Differently, d-WBS performed 12 months after ^131^I ablation was negative in all patients. t-WBS performed after empiric ^131^I therapy was negative in 59 patients and positive in 41. Among patients with positive scan, 27 presented local recurrence and 14 distant metastases (7 lung metastases, 4 bone metastases, and 3 both). Table 1 shows the characteristics of patients according to response to empiric ^131^I therapy after 12 months of treatment. Of all patients, 62 were in CR, 16 in PR, 8 in SD, and 14 showed PD. CR was evident in 67% of papillary DTC and in 37% of follicular DTC, PR in 17% of papillary DTC and in 13% of follicular DTC, SD in 7% of papillary DTC and in 13% of follicular DTC, PD in 10% of papillary DTC, and in 37% of follicular DTC (*P* < 0.01). A significant association (*P* < 0.001) between initial staging and response to empiric therapy was found, with the majority (83%) of patients in earlier stage (i.e., I and II) showing either CR of PR. Similarly, the response to empiric therapy was significantly (*P* < 0.01) associated with the initial ATA patients risk classification: 81% (35/43) of patients in low risk class showed either CR or PR. Pre-therapy Tg was higher in PD than in CR patients (Table 1). There was no statistical difference in the percentage of patients showing CR, PR, SD, and PD between negative and positive t-WBS (69, 15, 3, and 12% vs. 51, 17, 15, and 17%, respectively). However, when positive t-WBS were subdivided according to site of uptake, 89% of those with local recurrences showed either CR or PR, while 71% of patients with distant metastases at t-WBS were in SD or PD (Table 1), and this difference was statistically significant (*P* < 0.001). At multivariate logistic analysis, only these results of t-WBS were significant determinants of ^131^I therapy response, either CR or PR (odds ratio 0.32, 95% confidence interval 0.17–0.62, *P* < 0.001) (Table 2).

**Table 1:** Characteristics of patients according to 131-I therapy response.

	**CR (*n* = 62)**	**PR *(n* = 16)**	**SD (*n* = 8)**	**PD (*n* = 14)**	***p*-value**
Age (years)	42 ± 16	53 ± 16	46 ± 19	52 ± 15	0.06
Female gender (*n*)	41	10	6	11	0.7
**HISTOLOGY**					
Papillary (*n*)	56	14	6	8	<0.01
Follicular (*n*)	6	2	2	6	
**STAGE**					
I	45	6	3	6	<0.001
II	1	6	1	2	
III	12	3	2	6	
IV	4	1	2	0	
**ATA RISK GROUP**					
Low	24	11	2	6	<0.01
Intermediate	28	3	2	8	
High	10	2	4	0	
Time interval between therapy (months)	24 (12–240)	24 (12–96)	27 (12–96)	25 (12–96)	0.5
Pre-therapy Tg level (ng/ml) off^a^	13.5 (5–6,000)	56.8 (8–2,107)	60.5 (12–1,000)	62.2 (5–500)	<0.001
Administered 131-I activity (GBq)	3.7 (3.7–7.4)	5.2 (3.7–7.4)	5.5 (3.7–7.4)	5.2 (3.7–7.4)	0.09
**POST-THERAPY WBS**					
Negative	41	9	2	7	<0.001
Local disease	18	6	2	1	
Distant metastases	3	1	4	6	

*Data are presented as mean ± SD or median (range)*.

a *off L-thyroxine therapy*.

**Table 2:** Univariate and multivariate analysis of predictors of complete and partial remission after empiric 131-I therapy.

	**Univariate**		**Multivariate**	
	**Odds ratio (95% CI)**	***p*-value**	**Odds ratio (95% CI)**	***p*-value**
Age	0.39 (0.14 – 1.06)	0.06		
Female gender	0.56 (0.18 – 1.67)	0.06		
Histology (papillary)	0.18 (0.06 – 0.58)	0.005		
Tg pre-therapy off L-thyroxine therapy	1.0 (0.99 – 1.01)	0.8		
Stage (I and II vs. III and IV)	0.41 (0.16 – 1.10)	0.08		
ATA risk class (low vs. intermediate and high)	0.70 (0.26 – 1.18)	0.5		
Time interval between therapy	1.0 (0.99 – 1.01)	0.7		
Administered activity	1.0 (0.99 – 1.01)	0.8		
t-WBS (positive)	0.39 (0.15 – 1.02)	0.06		
t-WBS (distant metastases)	0.32 (0.17 – 0.62)	<0.001	0.32 (0.17 – 0.62)	<0.001

*CI, confidence interval*.

**Figure 2:**
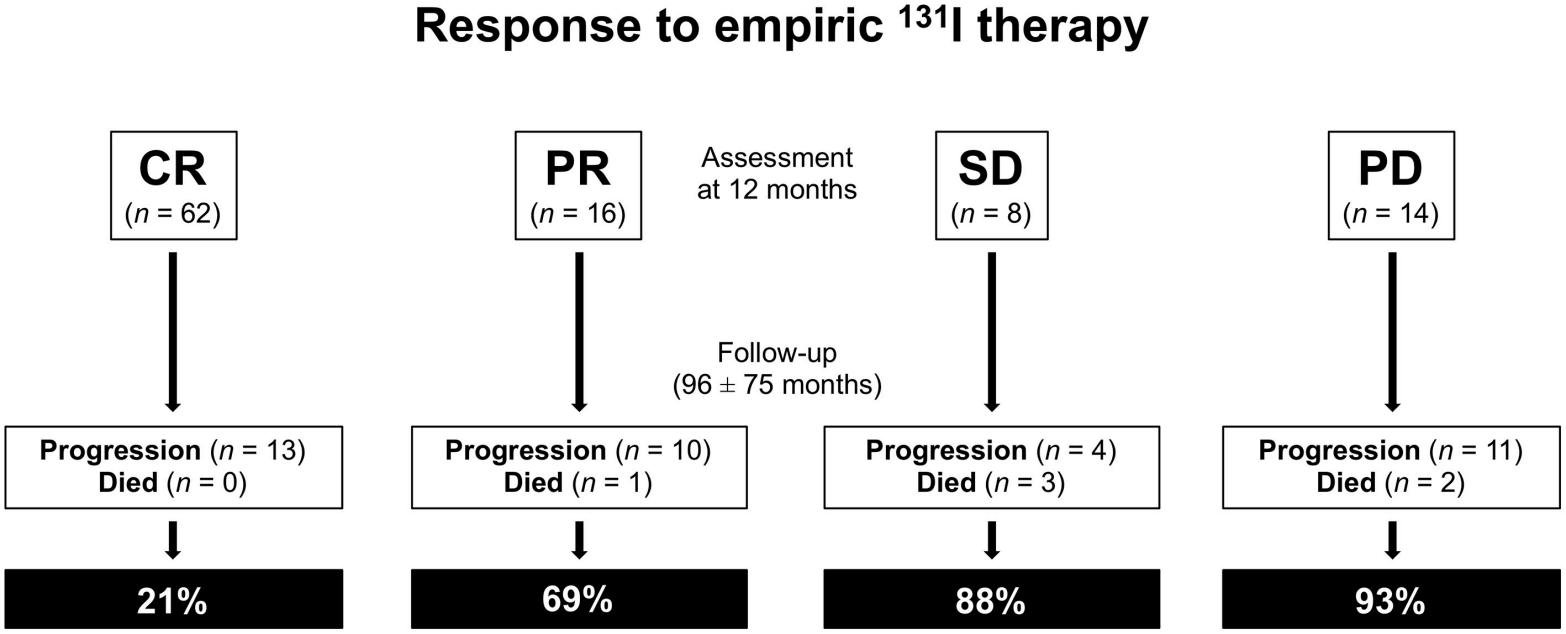
Response to empiric ^131^I therapy: assessment at 12 months and subsequent follow-up. CR, complete remission; PR, partial remission; SD, stable disease; PD, progression disease.

During subsequent follow-up (mean 96 ± 75 months, range 12–276) 38 patients showed progressive disease or relapse and 6 died of thyroid cancer (Figure 2). Of the 38 patients with progressive disease or relapse, 13 (34%) were in CR, 10 (26%) in PR, 4 (11%) in SD, and 11 (29%) had PD at 12 months evaluation after empiric 131-I therapy (*P* < 0.0001). Four of these 38 patients underwent surgery for lymph node recurrence and 34 further therapies with ^131^I (one additional therapy in 18 patients, two additional therapies in 11 patients, and three or more therapies in 9 patients). Of these latter 34 patients, 18 were retreated for PD (positive t-WBS after empiric ^131^I therapy) and 16 for persistent high Tg levels. Of the 38 patients with progressive disease or relapse 17 (45%) were in stage I, 9 (24%) in stage II, 8 (21%) in stage III, and 4 (10%) in stage IVa (p < 0.05). Moreover, of the same 38 patients 7 (18%) were in the ATA high risk group, 12 (32%) in the intermediate risk group, and 19 (50%) in the low risk group (*P* = 0.06). Of the 6 patients who died, 1 showed PR, 2 showed PD, and 3 were in SD at 12 months evaluation after empiric therapy (*P* = 0.6). Of the same 6 patients, 2 (33%) were in stage I, 3 (50%) in stage III, and 1(17%) in stage IV (*P* = 0.6). Also, 5 (83%) patients were in the ATA intermediate risk group and 1 (17%) in the high-risk group (*P* = 0.2).

**Figure 3:**
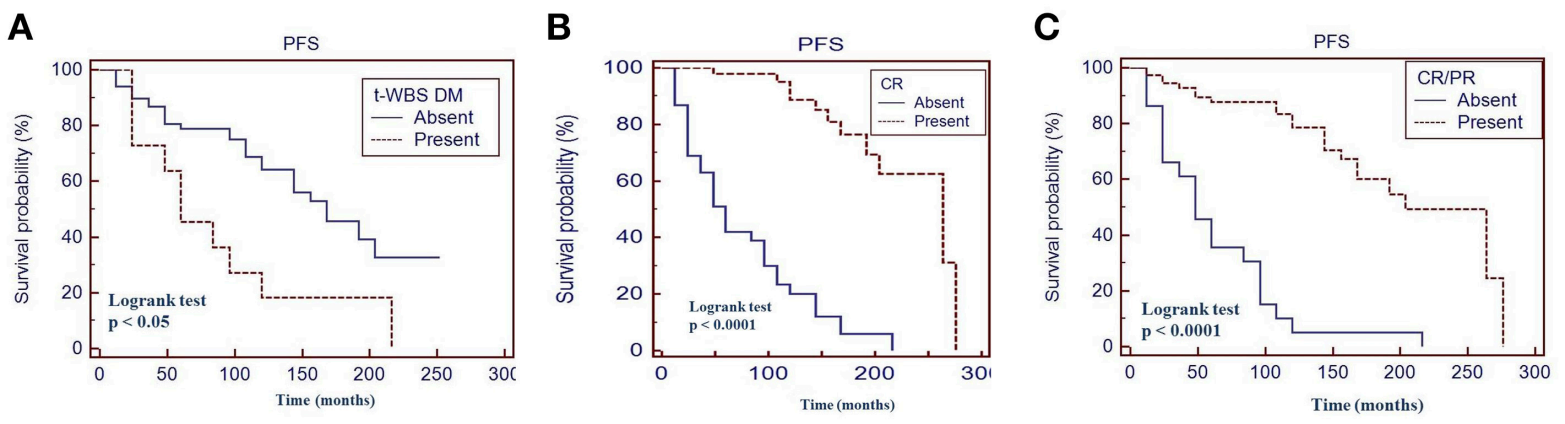
Progression-free survival (PFS) by Kaplan-Meier analysis and log-rank test based on the presence of distant metastases at therapeutic ^131^I whole body scan (WBS) **(A)** complete response at 12 months evaluation after ^131^I therapy **(B)** complete response + partial response at 12 months evaluation after ^131^I therapy **(C)**. t-WBS, therapeutic ^131^I WBS; DM, distant metastases; CR, complete remission; PR, partial remission.

**Figure 4:**
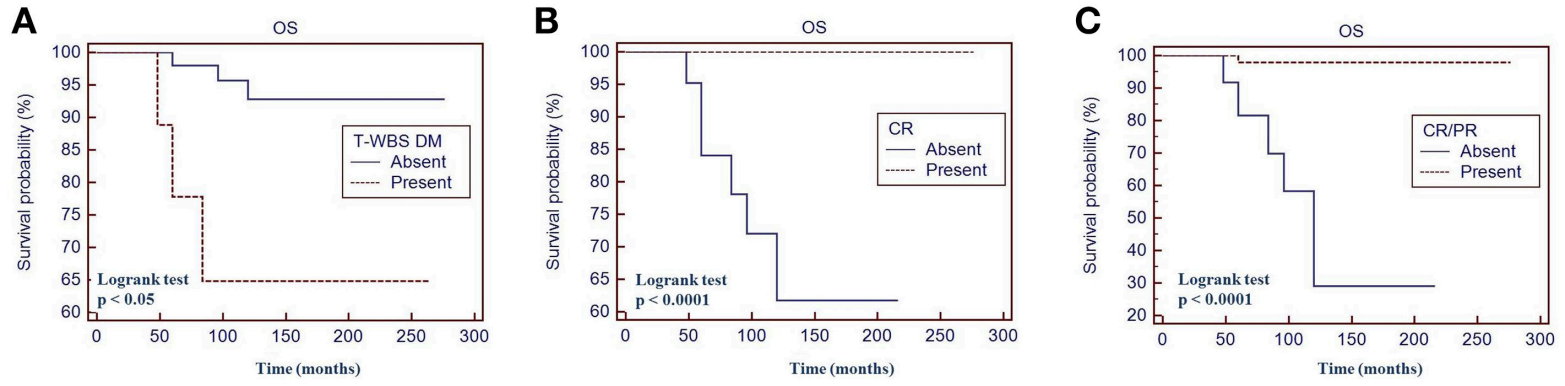
Overall survival (OS) by Kaplan-Meier analysis and log-rank test based on the presence of distant metastases at therapeutic ^131^I whole body scan WBS **(A)** complete response at 12 months evaluation after ^131^I therapy **(B)** complete response + partial response at 12 months evaluation after ^131^I therapy **(C)**. t-WBS, therapeutic 131-Iodine WBS; DM, distant metastases; CR, complete remission; PR, partial remission.

Fifty percent of patients with a positive t-WBS and 32% of those with a negative t-WBS showed progressive disease or relapse (*P* < 0.05). In particular, 46% of patients with regional uptake at t-WBS and 73% of those showing distant metastases had progressive disease or relapse. Of the 6 patients who died during follow-up, none was in CR at 12 months evaluation after empiric therapy and 4 had a positive t-WBS (3 with distant metastases). At univariate analysis, distant metastases (*P* < 0.05), CR (*P* < 0.0001) and CR + PR (*P* < 0.0001) were predictors of PFS (Table 3). Figure 3 shows the PFS at Kaplan-Meier analysis according to presence of distant metastases, CR and CR + PR. At multivariate analysis, only CR was retained in the model (*P* < 0.0001). At univariate analysis, distant metastases (*P* < 0.05), CR (*P* < 0.0001), and CR + PR (*P* < 0.0001) were significant predictors of OS (Table 3). Figure 4 shows the OS at Kaplan-Meier analysis according to presence of distant metastases, CR and CR + PR. At multivariate analysis, only CR or PR was retained in the model (*P* < 0.0001).

**Table 3:** Univariate predictors of progression free survival and overall survival based on clinical and imaging data.

	**Progression free survival**		**Overall survival**	
	**χ^2^**	***p*-value**	**χ^2^**	***p*-value**
Age	1.0	0.3	0.1	0.9
Female gender	0.9	0.3	0.2	0.7
Histology (papillary)	1.5	0.2	0.7	0.5
Tg pre-therapy off L-thyroxine therapy	0.1	0.9	0.1	0.8
Stage (I and II vs. III and IV)	1.9	0.2	3.8	0.5
ATA risk class (low vs. intermediate and high)	0.2	0.6	8.1	<0.01
Time interval between therapy	0.9	0.7	0.4	0.5
Administered activity	1.9	0.2	0.4	0.5
t-WBS (positive)	3.3	0.06	2.3	0.1
t-WBS (distant metastases)	4.9	<0.05	5.1	<0.05
Complete remission	48.9	<0.0001	15.8	<0.0001
Complete remission + partial remission	32.0	<0.0001	15.5	<0.0001

## Discussion

In this retrospective follow-up study of patients treated with ^131^I empiric therapy on the basis of elevated Tg, post-therapy WBS was positive in 41% of the patients and complete biochemical remission 12 month after ^131^I empiric therapy was observed in 62% of the entire population. Results of therapy were not related to age and gender, while patients with lower values of pre-therapy Tg achieved more frequently CR. Moreover, remission was more probable in patients in earlier stage of disease at presentation and in those in low-risk class as well as in patients not showing distant metastases at t-WBS performed after empiric ^131^I therapy. Both PFS and OS were better in patients showing remission at 12 months evaluation after empiric therapy as in those not showing distant metastases at t-WBS performed after empiric therapy.

According to ATA guidelines, ^131^I therapy is suggested in patients with elevated serum Tg levels when imaging did not show a tumor source amenable to directed therapy (6). While more than 50% of patients treated do have a reduction in Tg values (10, 16–18), improved survival has not been demonstrated (11–13). A large variability in percentage of patients showing positive t-WBS (ranging from 43 to 94%) has been reported, with a mean value of 61% (10, 19). On the other hand, the percentage of patients treated with ^131^I showing Tg values significantly decreased or undetectable Tg ranges from 20 to 72%, with a mean of 56% (10). No statistically significant difference in CR between patients with positive and negative t-WBS has been previously reported (11, 13, 16).

The results of our study confirm that 131-I therapy administered on the basis of elevated Tg is effective independently from the results of t-WBS. However, it should be noted that while 67% of patients with loco-regional uptake had CR, only 21% of those showing distant metastases achieved CR. It has been reported that in patients with structurally identifiable metastatic DTC ^131^I therapy failed to cause disease regression or conversion from progressive to SD in any patient (20). Moreover, serum Tg levels after ^131^I therapy decreased more frequently in patients with micro-metastases than in those with macro-metastases (12, 17), and CR has been observed only in 24.2% of patients with lung metastases (21).

The rationale of empiric ^131^I therapy is both diagnostic and therapeutic. However, it has been reported a relatively low rate of detection of pathological uptake mainly in patients with negative WBS at ablation (22, 23). The percentage of patients with positive t-WBS in our study is 41%, in the range reported in the literature (9, 10), and it should be noted that in our study the results of t-WBS at ablation have not been considered among the inclusion criteria, while a negative d-WBS after ^131^I ablation was one of the inclusion criteria.

No definite data on outcome of patients treated with ^131^I therapy on the basis of elevated Tg are present in the literature (11–13). In 42 patients with negative WBS and elevated Tg treated with a therapeutic dose of ^131^I and followed-up for 6.7 ± 3.8 years, Pacini et al. (11) found resolution of ^131^I uptake in 88.8% of lung metastases and in 61.1% of cervical node metastases. In the subgroup of patients with negative t-WBS (*n* = 12) one patient died (11). Fatourechi et al. (12) showed in 24 consecutive patients treated with ^131^I therapy (follow-up 6–33 months) that progression was evident in 13 patients with macro-metastases and 5 died of thyroid cancer, while the disease remained stable in 7 patients with micro-metastases. However, no statistical survival analysis was performed in these studies (11, 12). In 56 patients with DTC with negative WBS and increased serum Tg, van Tol et al. (13) found a 5-year survival of 100% in the group with positive t-WBS and of 76% in the group with negative t-WBS (*P* < 0.001). It should be noted that in that study a higher percentage of patients with positive t-WBS reached remission as compared to those with negative t-WBS (64 vs. 36%, *P* = 0.06) (13).

In our study we evaluated long-term follow-up in a group of 100 patients. Our results show a better PFS in patients without distant metastases at t-WBS and in patients showing CR or PR at 12 months evaluations after ^131^I therapy; moreover, OS was better in patients without distant metastases at t-WBS and in patients showing CR or PR at 12 months evaluations after ^131^I empiric therapy. Since the need for additional therapy was one of the end points for PFS, it is not surprising to find the presence of distant metastases at t-WBS to be a significant predictor. On the other hand, also OS is predicted by the presence of distant metastases at t-WBS.

Besides its retrospective observational nature, our study had other limitations. A similar control cohort in which ^131^I therapy was not performed is not available. Moreover, in a retrospective analysis the ability to discern certain treatment effects is limited by small sample size and limited number of events. Therefore, a randomized controlled study, with a larger cohort of patients, is needed to assess the prognostic impact of the empiric ^131^I therapy in patients with raised Tg values.

## Conclusions

The findings of this study suggest a beneficial effect of ^131^I therapy on the outcome of patients treated on the basis of elevated Tg value. However, survival is strongly affected by the achievement of remission, either complete or partial, at 12 months evaluation after ^131^I therapy and by the presence of distant metastases at t-WBS.

## Data Availability

The datasets generated for this study are available on request to the corresponding author.

## Ethics Statement

This study follows the principles expressed in the Declaration of Helsinki. All study participants waived informed consents owing to the retrospective analysis, and the study design was approved by the appropriate ethics review boards.

## Author Contributions

MK, LP, and AC contributed conception and design of the study. EM, GD, and TM collected the data and organized the database. LP and SL analyzed the data. MK and EZ wrote the first draft of the manuscript. All authors contributed to the final version of the manuscript.

### Conflict of Interest Statement

The authors declare that the research was conducted in the absence of any commercial or financial relationships that could be construed as a potential conflict of interest.

## References

[1] Ozata M Suzuki S Myamoto T Liu RT Fierro-Renoy F Degrot LJ. Serum thyroglobulin in the follow-up of patients with treated differentiated thyroid cancer. J Clin Endocrinol Metab. (1994) 79:98–105. 8027262 10.1210/jcem.79.1.8027262

[2] Duren M Siperstein AE Sheen W Duh QY Morita E Clark OH. Value of stimulated serum thyroglobulin levels for detecting persistent or recurrent differentiated thyroid cancer in high and low-risk patients. Surgery. (1999) 126:13–9. 10.1067/msy.1999.98849 10418587

[3] Pacini F Pinchera A Serum and tissue thyroglobulin measurements: clinical applications in thyroid disease. Biochimie. (1999) 81:463–7. 10.1016/S0300-9084(99)80096-0 10403176

[4] Mazzaferri EL Robbins RJ Spencer CA Braverman LE Pacini F Wartofsky L. A consensus report of the role of serum thyroglobulin as a monitoring method for low risk patients with papillary thyroid carcinoma. J Clin Endocrinol Metab. (2003) 88:1433–41. 10.1210/jc.2002-021702 12679418

[5] Pacini F Capezzone M Elisei R Ceccarelli C Tadei D Pinchera A Diagnostic 131-I whole body scan may be avoided in thyroid cancer patients who have undetectable stimulated serum Tg levels after initial treatment. J Clin Endocrinol Metab. (2002) 87:1499–501. 10.1210/jcem.87.4.8274 11932271

[6] Haugen BR Alexander EK Bible KC Doherty GM Mandel SJ Nikiforov YE. 2015 American thyroid *a*ssociation management guidelines for adult patients with thyroid nodules and differentiated thyroid cancer. Thyroid. (2016) 26:1–133. 10.1089/thy.2015.0020 26462967 PMC4739132

[7] Padovani RP Robenshtok E Brokhin M Tuttle RM. Even without additional therapy, serum thyroglobulin concentrations often decline for years after total thyroidectomy and radioactive remnant ablation in patients with differentiated thyroid cancer. Thyroid. (2012) 22:778–83. 10.1089/thy.2011.0522 22780333

[8] Yim JH Kim EY Bae KW Kim WG Kim TY Ryu JS. Long-term consequence of elevated thyroglobulin in differentiated thyroid cancer. Thyroid. (2013) 23:58–63. 10.1089/thy.2011.0487 22973946 PMC3539255

[9] Ma C Xie J Kuang A. Is empiric ^131^I therapy justified for patients with positive thyroglobulin and negative ^131^I whole-body scanning results? J Nucl Med. (2005) 46:1164–70. Available online at: http://jnm.snmjournals.org/content/46/7/1164.long 16000286

[10] Chao M. Management of differentiated thyroid cancer with rising thyroglobulin and negative diagnostic radioiodine whole body scan. Clin Oncol. (2010) 22:438–47. 10.1016/j.clon.2010.05.005 20561773

[11] Pacini F Agate L Elisei R Capezzone M Ceccarelli C Lippi F. Outcome of differentiated thyroid cancer with detectable serum Tg and negative diagnostic (131)I whole body scan: comparison of patients treated with high (131)I activities versus untreated patients. J Clin Endocrinol Metab. (2001) 86:4092–7. 10.1210/jcem.86.9.7831 11549631

[12] Fatourechi V Hay ID Javedan H Wiseman GA Mullan BP Gorman CA. Lack of impact of radioiodine therapy in Tg-positive, diagnostic whole-body scan negative patients with follicular cell-derived thyroid cancer. J Clin Endocrinol Metab. (2002) 87:1521–6. 10.1210/jcem.87.4.8373 11932275

[13] van Tol KM Jager PL de Vries EG Piers DA Boezen HM Sluiter WJ. Outcome in patients with differentiated thyroid cancer with negative diagnostic whole-body scanning and detectable stimulated thyroglobulin. Eur J Endocrinol. (2003) 148:589–96. 10.1530/eje.0.1480589 12773129

[14] Pace L Klain M Albanese C Salvatore B Storto G Soricelli A. Short-term outcome of differentiated thyroid cancer patients receiving a second iodine-131 therapy on the basis of a detectable serum thyroglobulin level after initial treatment. Eur J Nucl Med Mol Imaging. (2006) 33:179–83. 10.1007/s00259-005-1929-2 16205897

[15] Hovens GC Stokkel MP Kievit J Corssmit EP Pereira AM Romijn JA. Associations of serum thyrotropin concentrations with recurrence and death in differentiated thyroid cancer. J Clin Endocrinol Metab. (2007) 92:2610–5. 10.1210/jc.2006-2566 17426094

[16] Koh JM Kim ES Ryu JS Hong SJ Kim WB Shong YK. Effects of therapeutic doses of 131I in thyroid papillary carcinoma patients with elevated thyroglobulin level and negative 131I whole-body scan: comparative study. Clin Endocrinol. (2003) 58:421–7. 10.1046/j.1365-2265.2003.01733.x 12641624

[17] Kabasakal L Selcuk NA Shafipour H Ozmen O Onsel C Uslu I. Treatment of iodine-negative thyroglobulin positive thyroid cancer: differences in outcome in patients with macrometastases and patients with micrometastases. Eur J Nucl Med Mol Imaging. (2004) 31:1500–4. 10.1007/s00259-004-1516-y 15232654

[18] Sinha P Conrad GR West HC. Response of thyroglobulin to radioiodine therapy in thyroglobulin-elevated negative iodine scintigraphy (TENIS) syndrome. Anticancer Res. (2011) 31:2109–12. Available online at: http://ar.iiarjournals.org/content/31/6/2109.long 21737629

[19] Kalender E Umu Elboga U Çelen Y Demir H Sahin E Seyhan Karacavus S Empiric ^131^I treatment of high thyroglobulin levels in differentiated thyroid carcinoma after remnant ablation. Acta Med Mediterranea. (2014) 30:503–7. Available online at: http://www.actamedicamediterranea.com/archive/2014/medica-2/empiric-sup131supi-treatment-of-high-thyroglobulin-levels-in-differentiated-thyroid-carcinoma-after-remnant-ablation

[20] Sabra MM Grewal RK Tala H Larson SM Tuttle RM. Clinical outcomes following empiric radioiodine therapy in patients with structurally identifiable metastatic follicular cell-derived thyroid carcinoma with negative diagnostic but positive post-therapy 131I whole-body scans. Thyroid. (2012) 22:877–83. 10.1089/thy.2011.0429 22827641

[21] Song HJ Qiu ZL Shen CT Wei WJ Luo QY. Pulmonary metastases in differentiated thyroid cancer: efficacy of radioiodine therapy and prognostic factors. Eur J Endocrinol. (2015) 173:399–408. 10.1530/EJE-15-0296 26104753

[22] Kim WG Ryu JS Kim EY Lee JH Baek JH Yoon JH. Empiric high dose 131-iodine therapy lacks efficacy for treated papillary thyroid cancer patients with detectable serum thyroglobulin, but negative cervical sonography and 18F-fluorodeoxyglucose positron emission tomography scan. J Clin Endocrinol Metab. (2010) 95:1169–73. 10.1210/jc.2009-1567 20080852

[23] Rosario PW Mourao GF dos Santos JB Calsolari MR. Is empirical radioactive iodine therapy still a valid approach to patients with thyroid cancer and elevated thyroglobulin? Thyroid. (2014) 24:533–6. 10.1089/thy.2013.0427 24067080

